# Motivational Interviewing and Medication Review in Coronary Heart Disease (MIMeRiC): Intervention Development and Protocol for the Process Evaluation

**DOI:** 10.2196/resprot.8660

**Published:** 2018-01-30

**Authors:** Malin Johansson Östbring, Tommy Eriksson, Göran Petersson, Lina Hellström

**Affiliations:** ^1^ Pharmaceutical Department Kalmar County Council Kalmar Sweden; ^2^ eHealth Institute Department of Medicine and Optometry Linnaeus University Kalmar Sweden; ^3^ Department of Biomedical Science Malmö University Malmö Sweden; ^4^ Department of Clinical and Molecular Medicine Norwegian University of Sciences and Technology Trondheim Norway

**Keywords:** medication adherence, medication therapy management, pharmacist, coronary artery disease, quality of health care

## Abstract

**Background:**

Trials of complex interventions are often criticized for being difficult to interpret because the effects of apparently similar interventions vary across studies dependent on context, targeted groups, and the delivery of the intervention. The Motivational Interviewing and Medication Review in Coronary heart disease (MIMeRiC) trial is a randomized controlled trial (RCT) of an intervention aimed at improving pharmacological secondary prevention. Guidelines for the development and evaluation of complex interventions have recently highlighted the need for better reporting of the development of interventions, including descriptions of how the intervention is assumed to work, how this theory informed the process evaluation, and how the process evaluation relates to the outcome evaluation.

**Objective:**

This paper aims to describe how the intervention was designed and developed. The aim of the process evaluation is to better understand how and why the intervention in the MIMeRiC trial was effective or not effective.

**Methods:**

The research questions for evaluating the process are based on the conceptual model of change processes assumed in the intervention and will be analyzed by qualitative and quantitative methods. Quantitative data are used to evaluate the medication review in terms of drug-related problems, to describe how patients’ beliefs about medicines are affected by the intervention, and to evaluate the quality of motivational interviewing. Qualitative data will be used to analyze whether patients experienced the intervention as intended, how cardiologists experienced the collaboration and intervention, and how the intervention affected patients’ overall experience of care after coronary heart disease.

**Results:**

The development and piloting of the intervention are described in relation to the theoretical framework. Data for the process evaluation will be collected until March 2018. Some process evaluation questions will be analyzed before, and others will be analyzed after the outcomes of the MIMeRiC RCT are known.

**Conclusions:**

This paper describes the framework for the design of the intervention tested in the MIMeRiC trial, development of the intervention from the pilot stage to the complete trial intervention, and the framework and methods for the process evaluation. Providing the protocol of the process evaluation allows prespecification of the processes that will be evaluated, because we hypothesize that they will determine the outcomes of the MIMeRiC trial. This protocol also constitutes a contribution to the new field of process evaluations as made explicit in health services research and clinical trials of complex interventions.

## Introduction

### Evaluating Complex Health Care Interventions

Complex health care interventions consist of several components that interact or work independently of each other. Trials of complex interventions are often criticized for being difficult to interpret because effects of apparently similar interventions vary across studies dependent on context, targeted groups, and how the complex intervention was actually delivered [1]. The need for complexity is often questioned, since the effects of, or necessity for, the individual parts are seldom reported. Trials of complex interventions are also often difficult to replicate, both because the complexity might make them more sensitive to the context in which they are tested, and because interventions and designs are seldom reported in sufficient detail [1]. However, in the field of behavior change, such as medication adherence, complex interventions are often considered valuable because the determinants of behavior are multifaceted [1,2]. Guidelines for the development and evaluation of complex interventions have recently highlighted the need for better reporting of the development and evaluation of the intervention, including descriptions of how it is assumed to work, how these assumptions informed the process evaluation, and how the process evaluation relates to the outcomes evaluation [3-5]. The protocol for a randomized controlled trial (RCT) of a complex intervention aimed at improving pharmacological secondary prevention practice in coronary heart disease (CHD) is described in a separate paper, Motivational Interviewing and Medication Review in CHD (MIMeRiC) (forthcoming) [6]. In this paper, however, we describe the theoretical framework of the intervention, describe its development, and present the study protocol for our prespecified process evaluation, which will help explain the outcomes of the trial, inform about the generalizability of the trial’s results, and highlight barriers and facilitators that are important for successful implementation of the intervention.

### Theoretical Framework and Development of the Intervention

#### Patients’ Adherence Behavior

Adherence to medical treatment regimens is a complex act requiring both motivation and self-efficacy, and therefore nonadherence can be either intentional or unintentional [7]. Unintentional nonadherence occurs if the patient wants to adhere but is unable to because of difficulties with instructions, costs, remembering administration, or other practical reasons. Intentional nonadherence, on the other hand, occurs when the patient for some reason decides not to follow the recommendations. Factors influencing these types of adherence are different and need different management [8].

Patients’ adherence to medicine regimens is influenced by their attitude toward their medications; this can be measured with the instrument Beliefs about Medicines Questionnaire-Specific (BMQ-S) [9,10]. Beliefs are paramount determinants of both intentional and unintentional adherence and changes in beliefs have been linked to changed adherence behavior [9,11-15]. Patients with CHD develop more concern beliefs (ie, are more concerned) during the time after the event [16], which could explain the decrease in adherence in these patients [17,18].

There are several health psychology theories that describe what determines a behavior, and social cognitive theories have often been used to describe the behavior of (medication) adherence [7,19]. The health belief model, the theory of planned behavior, the reasoned action approach (RAA), and Bandura's social-cognitive model all share some ideas about how actual behavior follows from reasoning about expected outcomes of behavior, such as costs and benefits, and perceived control of a behavior (self-efficacy) [20]. According to the RAA, our behavior is determined by our intentions, and our intentions are determined by our attitude, perceived subjective norms, and perceived behavioral control. The subjective norm refers to a person’s belief about how important others will view their behavior, and this might be as important in determining intention as their own attitude toward their behavior. The perceived behavioral control refers to a person’s belief about their capacity to perform the behavior (very close to the concept of self-efficacy about the behavior), and this also influences their intention [20].

#### Motivational Interviewing

Motivational interviewing (MI) is a counseling approach used to elucidate a person’s motivation for change of a behavior [21,22]. MI recognizes people’s different readiness to change and how this should inform the support offered to a person, and it also recognizes the same determinants of behavior as the RAA. MI is a patient-centered approach which has been shown to be effective in different areas of health behavior change, including medication adherence [23,24]. MI is an approach relying on four main processes: engaging trust, focusing on the problems important to the client, evoking the client's own motivation and perceived resources, and planning specific actions leading to change [22]. All is done under the spirit of MI, which focuses on empathy, partnering with the patient, and emphasizing autonomy. Skills in MI such as affirmations, open-ended questions, and reflections are appropriate to elucidate the status of a patient’s medication use, to assess beliefs about medicines, subjective norms and perceived control, and to find the individual resources; all of these aspects are needed to influence the complex behavior of medication adherence. MI can be used to find the patient’s specific barriers to adherence and the patient's own solutions for both unintentional and intentional nonadherence, and MI offers a way of giving useful information in an intervention that relies on education about health and medicines. The use of MI or other cognitive methods in adherence interventions increases the likelihood of effect [25].

#### Secondary Prevention Quality

Despite established guidelines and widespread access to effective, inexpensive medicines, preventive treatment goals for blood pressure and cholesterol levels continue to be unmet for many coronary patients [26-28]. This is, in part, also an effect of patients’ resistance to the lifestyle changes needed to lower both blood pressure and cholesterol levels [29], but recent cohorts still indicate that the use of evidence-based drug treatments can be optimized to reach treatment targets [26]. Cardiac specialist nurses or pharmacists can help improve blood pressure management [30,31] and might also offer a model for other treatment targets in secondary prevention [32].

#### Medication Review

Medication review is a structured evaluation of a patient’s medicines with the aim of optimizing medication use and improving health outcomes. This entails detecting drug-related problems (DRPs) and recommending interventions [33]. Medication reviews have been evaluated in different settings such as hospitals, care homes, and primary care [34-36], but the diversity of methods and outcomes makes comparison and meta-analyses difficult. However, recommendations are to conduct future trials in high-risk populations, with professionals allowed to change patient medication, and with long-term follow-up [34]. Side effects of drugs are common DRPs and identification of side effects is essential to balance the harms and benefits of secondary prevention medications that are often used for the rest of life. Medication review has been used as part of an integrated medicines management model (LIMM, Lund Integrated Medicines Management) [37,38], which was used as the model for our intervention. We aimed to adapt the LIMM to suit the care process for CHD: a care process characterized by a short hospital stay, polypharmacy initiated in hospital, and follow-up of effects and side effects as outpatients.

#### Theoretical Framework for Intervention Design

An adherence intervention should be based on what is known about adherence behavior [2,8]. The reasons for nonadherence are multiple and individual, and therefore an intervention must have a broad inventory phase and an individualized problem-solving phase to be effective in a wide group of patients. Our theoretical framework was based on the adherence model described by World Health Organization (WHO), which comprises five dimensions (see Table 1) [8]. The model, which is based on theory and evidence about adherence behavior, formed the basis of how we intend the intervention to work in terms of adherence. We added quality of treatment as one of our intervention targets since a need to improve the quality of pharmacological secondary prevention has been previously highlighted [27,28,39]. It is our core understanding that an ambition to increase adherence to treatment should always be accompanied by an evaluation of the treatment itself, so as not to improve adherence to a treatment that might be harmful. In this way, our theoretical framework for the intervention reflects the concept of pharmaceutical care described by Cipolle and Strand [40]: “Pharmaceutical care practitioners accept responsibility for optimizing all of a patient’s drug therapy, regardless of the source…, to achieve better patient outcomes and to improve the quality of each patient’s life. This occurs with the patient’s cooperation and in coordination with the patient’s other health care providers.”

The basis of the intervention is that all dimensions should be covered in an inventory of the patient’s drug-related needs, and that activities are subsequently undertaken in the dimensions where problems have been identified.

#### Conceptual Model of Change

On the basis of the adherence model by the WHO, the RAA as a more general model for predicting behavior, and our understanding of how prescribing treatment determines outcomes, we made a conceptual model of how the change processes of the intervention would act to change the outcomes we set out in our RCT [6]. The model in Multimedia Appendix 1 describes how the optimized outcomes follow from full adherence to the optimal treatment, and how the intervention acts by two methods to improve these variables. MI is thought to act on the adherence variable and the medication review on the treatment variable. The intervention can only affect the outcomes if a patient has a problem with adherence (current or expected in the future) and/or treatment quality.

#### Pilot Evaluation: Changes Made From Pilot to RCT

In a pilot study in 2012, we tested an intervention based on MI and medication review aimed at improving patients’ beliefs about medicines and adherence to secondary prevention of cardiovascular disease [41]. The pilot RCT of 21 patients resulted in insights regarding the feasibility of the intervention and study design. Patients with more negative beliefs (BMQ-S) changed toward more positive beliefs, but there was no difference between groups at follow-up. A need to stratify the randomization based on baseline beliefs was identified [41]. We tested a method of categorizing patients according to their beliefs in the pilot study population and found that half of the population (10 patients) had an attitude with a potentially negative impact on adherence [42].

In line with the recommendation from the Medical Research Council (MRC) [3], we have also made a qualitative evaluation of the pilot study (not published). Interviews of 8 patients showed that, overall, the patients were positive about the intervention and felt more informed by it. The interviews also informed us that some patients changed their views on medicines in a positive direction, although it was not enough to categorize them to an attitude group with higher adherence. The care processes for these 8 patients were also compared with the theoretical framework of the intervention, and we found that all the patients were affected by some part of the intervention; patients with a negative attitude toward their medicines were mainly affected in the adherence dimensions, whereas patients with a positive attitude were helped in the area of quality of treatment. However, our evaluation showed that not all the dimensions of adherence were well covered in the identification phase of the intervention and that the pharmacist felt the need for more training in MI.

**Table 1:** 

Target of intervention and dimensions to influence		Activities	
**Adherence**			
	Social/economic factors		Reviewing patients' need for social support
			Referring to support group
	Health system/ Health-care team factors		Adding time and competence to the HCT team
			Building good patient-provider relationship
			More patient-provider contacts
	Condition-related factors		Identifying and solving other health problems that might affect adherence, ie, depression, stress, pain, pulmonary disease
	Therapy-related factors		Simplifying regimen
			Minimizing side effects
			Patient-tailored prescriptions
			Continuous monitoring and reassessment of treatment and adherence
	Patient-related facators		Mutual goal setting
			Changing the patient's attitude towards medicine-taking, changing beliefs
			Supporting patient's self-efficacy in medicine-taking
			Memory aids and reminders
**Quality of treatment**			
	Appropriate medication		Change of prescribing if: Medicines without indication Untreated conditions
	Effective medication		Change prescribing if: Not prescribed according to guidelines Unmet treatment goals
	Safe medication		Change prescribing if: Any clinical manifestations are due to adverse drug reaction Risk for future adverse drug reactions

^a^Five dimensions of adherence, World Health Organization's model for adherence [8].

^b^Pharmaceutical care according to Cipolle and Strand [40].

These results from the qualitative evaluation led us to the following three decisions about the intervention design: (1) all patients should be targeted for intervention regardless of their baseline attitude toward medicines, (2) the intervention needs to be intensified or prolonged for patients with a negative attitude, and (3) a focus on training and applying MI as the basis of the intervention is required.

We also added a second patient consultation in our intervention design before setting up the RCT. This decision was partly based on the experiences of cardiology nurses in another study with prolonged follow-up for this group of patients (not yet published). In the standard care period, patients are discharged from hospital 3 days after admission for an acute event or the day after a planned coronary intervention. At discharge, they receive prescriptions for their secondary prevention medicines to cover 12 months. Patients normally have a follow-up appointment at the cardiology clinic about 2 months after discharge, which includes an evaluation of effects and any side effects of the medicines. Until this follow-up the cardiology clinic is always responsible for the patient’s treatment. Most patients with no need for further adjustments are referred to primary care after their follow-up meeting, but they are rarely summoned for a visit by their primary care facility. In Sweden, patients need new prescriptions after 12 months, and our experience is that patients with little previous contact with their primary care facility often feel unsure about where to turn when they need new prescriptions or have questions about future treatment (qualitative interview study of 18 patients with CHD, not yet published).

Psychological recovery from acute myocardial infarction has been described as occurring in 4 stages, from the acute phase of accepting what has happened to the last stage of living again, or being back to normal [43]. The recovery process can take up to 6 months, and we theorize that the intervention needs to follow the patient until this stage of normality occurs if secondary prevention treatment is to be part of normality. Therefore, we decided to add a consultation at 10 months after discharge to meet all patients when fully recovered, and as a way of supporting the transition from cardiac specialist care at the hospital to the primary care facility.

### Protocol for the Process Evaluation

#### Framework of the MIMeRiC Trial Parallel Process Evaluation Protocol

As recommended by the MRC guidelines for developing and evaluating complex interventions [3], a process evaluation should be conducted to “explain discrepancies between expected and observed outcomes, to understand how context influences outcomes, and to provide insights to aid implementation”. Grant et al, who proposed a framework for the design of process evaluations, highlighted that the purpose of the process evaluation should be explicitly placed along with the original research questions, and that the processes that are not evaluated should be acknowledged [4]. They also proposed a model of prespecified evaluations to quantitatively examine prior hypotheses about trial processes, although post hoc evaluations have the advantage of being flexible for examining unexpected findings. This study includes a protocol for the prespecified evaluation. The logic model of how the intervention is expected to work (Multimedia Appendix 1) underlies our design of the process evaluation [3,4].

Multimedia Appendix 2 shows in detail how we expect the two methods, MI and medication review, to act on different determinants of the adherence and prescribing variables. We intend the MI part to be able to assess determinants such as perceived effects and side effects, beliefs, skills, and values, and also to act on these. In this way, we intend the intervention to work on the adherence variable through an effect on how the patients feel (how they perceive effects and side effects), reason (their values, risk perceptions, and beliefs about medicines and adherence), and act (their skills). The medication review is intended to affect the prescribing variable by increasing physicians’ knowledge about the patient and the guidelines. The action on prescribing will also affect DRPs experienced by the patient, which in turn is a determinant of adherence.

When designing the trial, we included process outcomes related to the medication review, that is, the number of DRPs found and quality of prescribing. At that time, we were also informed that it was essential to have some quality control of the MI performance to be a relevant trial in this research field. Therefore, we had prepared for this data collection before the trial started. After the start of the trial and inspired by the aforementioned guidelines, we decided on another four process outcomes that would help us understand any effect of the intervention or any lack of effect [3,4]. Those outcomes are related to cardiologists’ and patients’ experience of the intervention, patients’ beliefs about medicines, and patients’ experience of the follow-up care as a whole.

These processes are thought to affect patient adherence and doctors’ prescribing, which in turn are thought to affect the trial outcomes (treatment goal attainment, health-related quality of life, and hospital care need) (Multimedia Appendix 2).

#### Study Design of the RCT in Brief

The MIMeRiC trial is an RCT with 2 parallel groups (N=418) and 12 months follow-up. Patients with CHD identified and followed at the cardiology clinic of Kalmar County Hospital are randomized to usual care (control) or usual care plus a follow-up program with medication review and MI. Ethical approval has been obtained from the Regional Ethics Committee, Linköping (Dnr-2013/236-31). The trial (clinicaltrials.gov, NCT02102503) has been fully described in the forthcoming protocol [6]. Patients in the intervention group meet a clinical pharmacist at the cardiology clinic 2 to 5 times during the year after discharge depending on need, and problems with adherence or prescribing are solved in collaboration with the patient and/or the cardiologist.

The primary outcome of the trial is the proportion of patients reaching the treatment goal for low-density lipoprotein cholesterol. Secondary outcomes involve the effects on blood pressure, patient adherence, quality of life, and health care use. An economic evaluation of the intervention is also planned.

#### Aim and Research Questions

The aim of this process evaluation is to better understand how and why the intervention was effective or not effective.

Research questions include the following:

1. What was actually delivered in the medication review (DRPs solved and results documented)?
2. How did the cardiologists experience the involvement of and interaction with a clinical pharmacist?
3. Was MI used consistently with MI principles?
4. Did the intervention change how the patients felt, reasoned about, or acted toward their cardiovascular medicines?
5. Did the intervention change the patients’ beliefs about medicines (before vs after, and between groups)?
6. How did the intervention affect the patients’ experience of their follow-up care after CHD?
7. Did the intervention change the quality of prescribing?
8. Did the intervention change the patients’ adherence? This is an outcome measure in the MIMeRiC trial and is described in Multimedia Appendix 2 only for consistency.

In Multimedia Appendix 2, the research questions are shown in relation to the conceptual model to show which intervention processes are not evaluated in this study.

#### Management and Governance

The process evaluation is to be conducted in parallel with the MIMeRiC trial; some data will be collected together for the two studies. Data analysis for the two studies will also be conducted in parallel. The same researchers are responsible for the RCT, delivering the intervention, and planning and conducting the process evaluation; our small research team and restrictive funding did not allow for any independent evaluators to be involved [3].

Most of the research questions are covered by the ethical approval obtained for the RCT, but a supplemental ethical approval was granted for qualitative study of the patients’ experience of the intervention (interviews) and their views on the follow-up care after discharge (questionnaire).

## Methods

### Overall Study Design

The process evaluation is a mixed-method study. Three research questions will be studied using qualitative methods, three using quantitative methods, and one using a mix of qualitative and quantitative methods. This was based on the MRC guidelines: “Hence, when feasible it is often useful to combine quantitative data on key process variables from all sites or participants with in-depth qualitative data from samples purposively selected along dimensions expected to influence the functioning of the intervention” [3].

### Methods for Research Question 1: What Was Actually Delivered in the Medication Review?

This will be studied using a descriptive, quantitative method. The number and type of DRPs will be described using the 7 categories suggested by Cipolle and colleagues: (1) adverse drug reaction, (2) ineffective drug, (3) need for additional drug therapy, (4) dosage too low, (5) dosage too high, (6) unnecessary drug therapy, and (7) noncompliance. If acting on the DRPs has any effects that are documented in the electronic health record (EHR), these will also be described. Data are collected from the pharmacists’ documentation in the EHR and from separate study notes about DRPs that pharmacists record during the intervention. Data on the effects of actions on DRPs will be collected from the EHR; the pharmacists’ documentation will be used with laboratory results, documentation by primary care or other professionals at the cardiology clinic when relevant.

### Methods for Research Question 2: How Did the Cardiologists Experience the Involvement of and Interaction With a Clinical Pharmacist?

This question will be answered by a qualitative questionnaire to cardiologists with 4 questions: (1) What is your opinion about the patients having a consultation with a pharmacist to discuss their medicine regimen, after the standard follow-up with a physician? (2) What is your opinion about the pharmacists conducting a medication review and contacting you as a cardiologist to consider their suggestions? (3) What is your opinion about the collaboration with the pharmacists in the study? (4) Would you like to add anything else?

Questionnaires will be issued at the end of the intervention period and answered anonymously by the cardiologists. Collected data will be analyzed with inductive content analysis.

### Methods for Research Question 3: Was the MI Used Consistently With MI Principles?

This will be a quantitative assessment of the integrity of MI delivered by the clinical pharmacists. All in-person consultations in the intervention group are audio-recorded if permitted by the patient. A random sample of these recordings, the number corresponding to 20% of in-person consultations, will be coded with the Motivational Interviewing Treatment Integrity behavioral coding system version 4.2.1 (MITI 4.2.1) [44] by an independent coding institute (the MIQA group at the Karolinska Institute, Stockholm). A randomly selected 20 min of these recordings will be coded; for one-third of the consultations, the first 20 min will be coded; for one-third, the middle 20 min will be coded; and for one-third, the last 20 min will be coded. Competency in MI will be described by four global scores: cultivating change talk, softening sustain talk, partnership, and empathy. In addition, the ratios of change talk to sustain talk and questions to reflections will be assessed. These scores and ratios will be related to recommended levels of MI competency. The target behavior of the MI consultations is defined as: start taking medicines regularly or maintain this behavior. Changing a patient’s attitude toward their medicines in a positive direction is a strategy for maintaining or reaching the target behavior of regularly taking the medicines. On the basis of our theoretical framework and our pilot evaluation, we assumed that patients with a negative attitude need the MI component of the intervention, and should have this, whereas accepting patients are considered adherent and the need for MI might be less obvious. These patients are targeted by the intervention partly to maintain their target behavior but primarily to solve other DRPs and to improve the quality of prescribing. However, there is no distinct line between these groups; problems with adherence can arise in the group with an accepting attitude as well. To ensure that both groups are equally represented in the assessment of consultations, we will stratify the samples so that 20% of consultations with patients with a negative attitude and 20% of consultations with patients with an accepting attitude are coded. For the latter group, it might be that DRPs make the target behavior undesirable for a particular drug and, if so, only two of the global scores—partnership and empathy—will be used. Such consultations without a target behavior will be valued on a scale for person-centeredness instead of MI competency. The analysis will inform about the frequency of consultations with a target behavior (among the random sample), the fidelity of MI, and the fidelity of person-centered consultations.

### Methods for Research Question 4: Did the Intervention Change How the Patients Felt, Reasoned About, or Acted Toward Their Cardiovascular Medicines?

This will be studied qualitatively by assessing how patients’ experiences of the intervention relate to the intended mechanisms of the intervention, as described in the conceptual model (Multimedia Appendix 2) and the framework for the process evaluation. For this question, the method of focus group interviews was chosen because it is an effective method of gathering information and is especially valuable in the evaluation of program experiences. The main advantage of focus groups over individual interviews is the richness and quality of the data that arise, because participants are listening to the answers of others. Comments might trigger memories and thoughts that would not come up in individual interviews, and participants’ comments on each other weed out false or extreme views [45].

Three focus group interviews will be carried out with 4 to 6 intervention patients in each group. The interview and discussion will be led by a moderator, a pharmacist who is familiar with the study but is not involved in the care of the patients. Because the intervention is primarily intended to affect beliefs and adherence among those who have a negative attitude and therefore a higher risk of nonadherence, the sample of patients chosen for the focus groups will be from patients with a negative attitude toward their medicines at baseline. With purposeful sampling among the patients who have taken part in the full intervention, we will try to cover both men and women, those newly diseased and those with a history of CHD, those with acute and those with chronic disease, and those who changed their attitude after the intervention in addition to those who did not.

Questions will be asked about how the patients experienced the consultation with the pharmacist and how they perceived that it had affected their medicine-taking behavior or their reasoning about medicines. The moderator will encourage the patients to describe all aspects by using probing questions. The moderator will also be supported by an observer whose main role will be to record all nonverbal communication in the focus groups. The focus group interviews will be audio-recorded and transcribed verbatim and then analyzed using deductive content analysis. A coding scheme of categories will be constructed based on the conceptual model about how the intervention is intended to work on adherence. The moderator will not be analyzing the interviews but will be accessible for questions about how to read the transcripts.

### Methods for Research Question 5: Did the Intervention Change the Patients’ Beliefs About Their Cardiovascular Medicines?

This will be studied primarily quantitatively, but certain aspects will also be illustrated qualitatively by the interviews with patients.

Data on beliefs about medicines will be collected in the MIMeRiC trial with the BMQ-S instrument administered 3 times: baseline (after the standard care follow-up), after 10 months (during the intervention), and after 15 months (after the intervention). In this study, we will ask the patients to consider only their heart medicines as they answer the BMQ-S. The instrument consists of two subscales, one for the perceived necessity for the drugs and one for perceived concerns about the drugs. The two subscales have 5 items each and are assessed by a 5-point verbal descriptor scale ranging from strongly disagree to strongly agree.

As instructed by the originators, numerals (1-5) will then be assigned to each statement of agreement to summarize each subscale with 5 to 25 points. The difference between the necessity and concern scales (range: −20 to 20) will then be related to the cost-benefit analysis for each patient’s medications. A positive difference means that the patient perceives the benefit of taking the drugs to outweigh the risks of taking the drugs, and the more positive the difference is, the more adherent the patient is supposed to be [9]. It has also been found that categorization based on high (>15) or low (<15) scores on each scale, yielding 4 categories (accepting, ambivalent, neutral, and skeptical) predicts adherence behavior [46,47]. Because the scales have ordered verbal categories, it is not correct to assign numerals and sum them to a global score, because the data are only ordered within the structure while the distance between the agreement statements or the magnitude of them is not known [48]. Even though the categorization also depends on the global sum of each scale, this is a more correct way of handling the data. Therefore, we chose this as our primary outcome in analyzing the effects of the intervention on beliefs. The stratified randomization of patients in the MIMeRiC trial was also based on these four categories.

In this study, we will:

1. analyze whether there is a difference between the control group and the intervention group in the proportion of patients in each category at follow-up (15 months)
2. describe temporal changes in the 4 categories over the 3 assessments in the control and the intervention groups
3. analyze changes in the sum of each subscale, and differences between the intervention and control groups
4. analyze changes in each item of the scales, and differences between the intervention and control groups
5. analyze the transcribed material from the focus group interviews with intervention patients described above using deductive content analysis methods, based on the 10 items of BMQ-S, as a way of including the results of the quantitative study of beliefs

While item 1 in the list is a primary objective, items 2 to 5 refer to secondary objectives.

Analyses 1 to 3 will be adjusted for variables thought to be influential: sex, age, type of CHD, and history of CHD. In our pilot study, most patients with an ambivalent attitude toward their drugs had a history of CHD [42].

### Methods for Research Question 6: How Did the Intervention Affect the Patients’ Experience of Their Follow-Up Care After CHD?

This is a qualitative study of how patients perceive their care after discharge from hospital following CHD. An open question will be enclosed with the questionnaires sent at the 15-month follow-up to all patients enrolled after January 1, 2016. The instructions to these patients will be: *Please take a moment to write freely about your view of the follow-up care you received after your myocardial infarction or angina*

### Methods for Research Question 7: Did the Intervention Change the Quality of Prescribing?

This will be studied using quantitative methods. There are several models for assessing the appropriateness of prescribing or medication use which have been used in intervention studies of medication reviews [49,50]. For this study with a selected group of patients with CHD, we chose to use a tool developed for this diagnosis: the Medication Assessment Tool for evaluation of secondary prevention of CHD (MAT-CHDsp) [51]. The tool is a summary of review criteria based on clinical guidelines and has been developed for use in clinical audits. Compared with the variables in the Swedish Quality Register SWEDEHEART, in which the proportion of patients with a prescription from a certain drug class is registered, MAT-CHDsp has a more individual application where prescriptions that do not follow the guidelines can be justified if the reason is stated in the EHR. The tool was updated in 2014 based on the European guidelines from 2011 and 2012 for myocardial infarction or acute coronary syndrome and the Swedish guidelines for heart disease from 2011 and was then validated in 22 patients in this study. MAT-CHDsp comprises 28 review criteria for which the assessor chooses *not applicable*, *yes*, *no*, *no information found*, or *no, but this is justified*. As an example, the treatment of a patient with CHD and no left ventricular (LV) dysfunction but with side effects from several statins documented in the EHR would be marked as *not applicable* for the criterion “Patient with CHD with LV dysfunction...is prescribed a beta-blocker” and *no, but justified* for the criterion “Patient with CHD is prescribed atorvastatin or simvastatin.”

The MAT-CHDsp will be applied to a random sample of 20% of the patients in each of the intervention and control group. Data on the prescribing 6 months after discharge will be collected retrospectively from the EHR at assessment. Applicable criteria will then be analyzed for adherence to guidelines or justified nonadherence. The results will be compared between the groups in terms of adherence and justified nonadherence, as a measure of the quality of the prescribing for secondary prevention [52].

### Integrating Results of Analysis

Some process evaluation questions will be analyzed before and others will be analyzed after the outcomes of the MIMeRiC RCT are known. This will be determined by time and availability of the data as we prioritize having the results of the RCT ready as soon as all data can be analyzed.

The 7 research questions for the process evaluation will be analyzed by their respective methods and then, when all are complete, the analysis will be combined and applied to the results of the MIMeRiC trial. This will help us understand whether any effects of the intervention are related to the concepts we used in the design. If appropriate, we may carry out additional analyses to test hypotheses generated from integration of the process evaluation data with the trial outcomes; an example of this would be an analysis of the effect of BMQ-S on adherence. The full report of the process evaluation will be published in a peer-reviewed journal and a summary of the findings of and cross-references to the main MIMeRiC trial will aid interpretation of the evaluation.

## Results

Collection of data has been ongoing as part of the MIMeRiC trial, and some process evaluation analyses can start during 2017. The MITI-coding of 64 consultations have been conducted, but the results are not yet analyzed. The method of focus groups and deductive content analysis have been piloted and found to be useful for the question about how patients experience the intervention, and more focus group interviews will be conducted in September 2017.

## Discussion

This paper describes the framework for the design of the intervention tested in the MIMeRiC trial, development of the intervention from the pilot stage to the complete trial intervention, and the framework and methods for the process evaluation. Providing the protocol of the process evaluation allows prespecification of the processes that will be evaluated, because we hypothesize that they will determine the outcomes of the MIMeRiC trial. This protocol also constitutes a contribution to the new field of process evaluations as made explicit in health services research and clinical trials of complex interventions. The two active parts of the intervention, motivational interviewing and medication review, are both quantitatively evaluated with their specific instruments: Motivational Interviewing Treatment Integrity (MITI) coding and categorization of all DRPs acted on, as well as assessment of secondary prevention treatment quality. Qualitative methods are used to inform about patients’ experiences of the intervention and to capture any unforeseen effects on the secondary care process experienced by patients.

A limitation might be that we are a small research team and have been the designers, implementers, as well as evaluators of this complex intervention. This may not be in line with the guidelines of the MRC. However, the guidelines for process evaluation of complex interventions issued by the MRC in 2015 have served us well in defining the requirements for properly evaluating our intervention process. We hope that this protocol can inspire other research teams to publish process evaluation protocols so that complex interventions in health services research in general, and medication adherence in particular, can be interpreted with more confidence in the future.

## Appendix

Multimedia Appendix 1 Conceptual model of change processes in the intervention. The variables we aim to affect are shown in orange, the goal of optimal treatment is shown in yellow, outcomes measured in the randomized controlled trial are shown in green, and the blue lines represent how the two accompanying parts of the intervention act on the variables. The adherence variable is determined by factors identified by the reasoned action approach (The prescribing variable is determined by adherence to guidelines and the level of individualization.). The consequences of inappropriate prescribing are shown in gray boxes; these interact with factors that influence adherence. CHD: coronary heart disease; EBM: evidence-based medicine.

Multimedia Appendix 2 Conceptual model of intervention change processes and what will be covered by the process evaluation. This figure shows more detail of how we expect the motivational interviewing and medication review to act on determinants of adherence and prescribing. More attention, a nonspecific part of any extra follow-up intervention, shown as a blue oval, is thought to act positively on patients' attitudes toward and subjective norms about adherence. Blue arrows indicate what will be assessed by the intervention and red arrows indicate what determinants we think can be influenced by it. The gray boxes contain the research questions for the process evaluation. CHD: coronary heart disease; EBM: evidence-based medicine.

## Abbreviations

BMQ-S: beliefs about medicines questionnaire-specific
CHD: coronary heart disease
DRP: drug-related problem
EHR: electronic health record
LIMM: LundIntegrated Medicines Management
LV: left ventricular
MAT-CHDsp: medication assessment tool for evaluation of secondary prevention of coronary heart disease
MI: motivational interviewing
MIMeRiC: motivational interviewing and medication review in coronary heart disease (trial)
MITI: motivational interviewing treatment integrity
MRC: Medical Research Council
RAA: reasoned-action approach
RCT: randomized controlled trial
WHO: World Health Organization
