# Supplementary figures and images for: Motivational Interviewing and Medication Review in Coronary Heart Disease (MIMeRiC): Intervention Development and Protocol for the Process Evaluation
Source: JMIR Res Protoc. 2018 Jan 30;7(1):e21. doi: 10.2196/resprot.8660 (PMC5811650; doi:10.2196/resprot.8660)

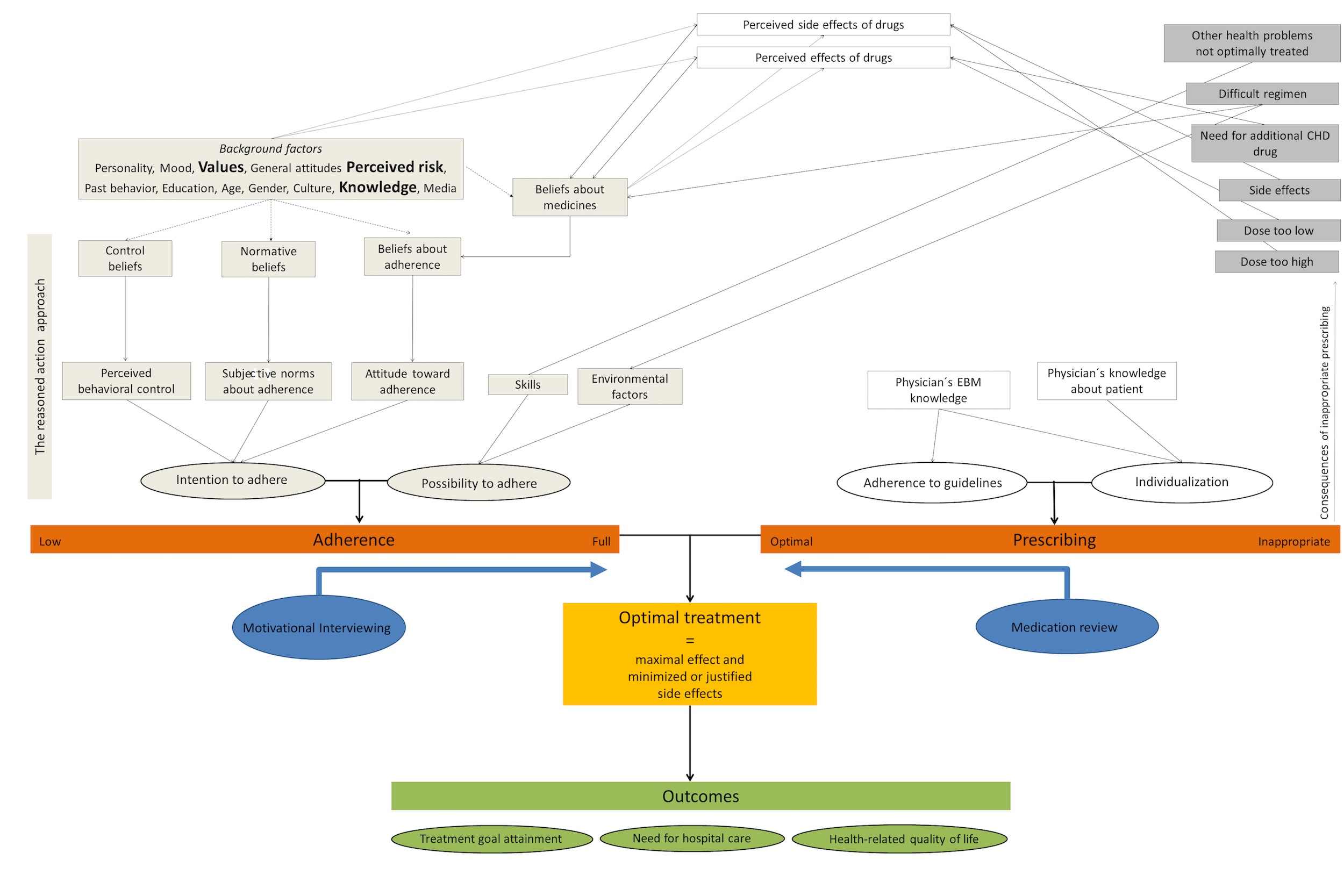

Supplement: Multimedia Appendix 1 [file resprot_v7i1e21_app1.png]

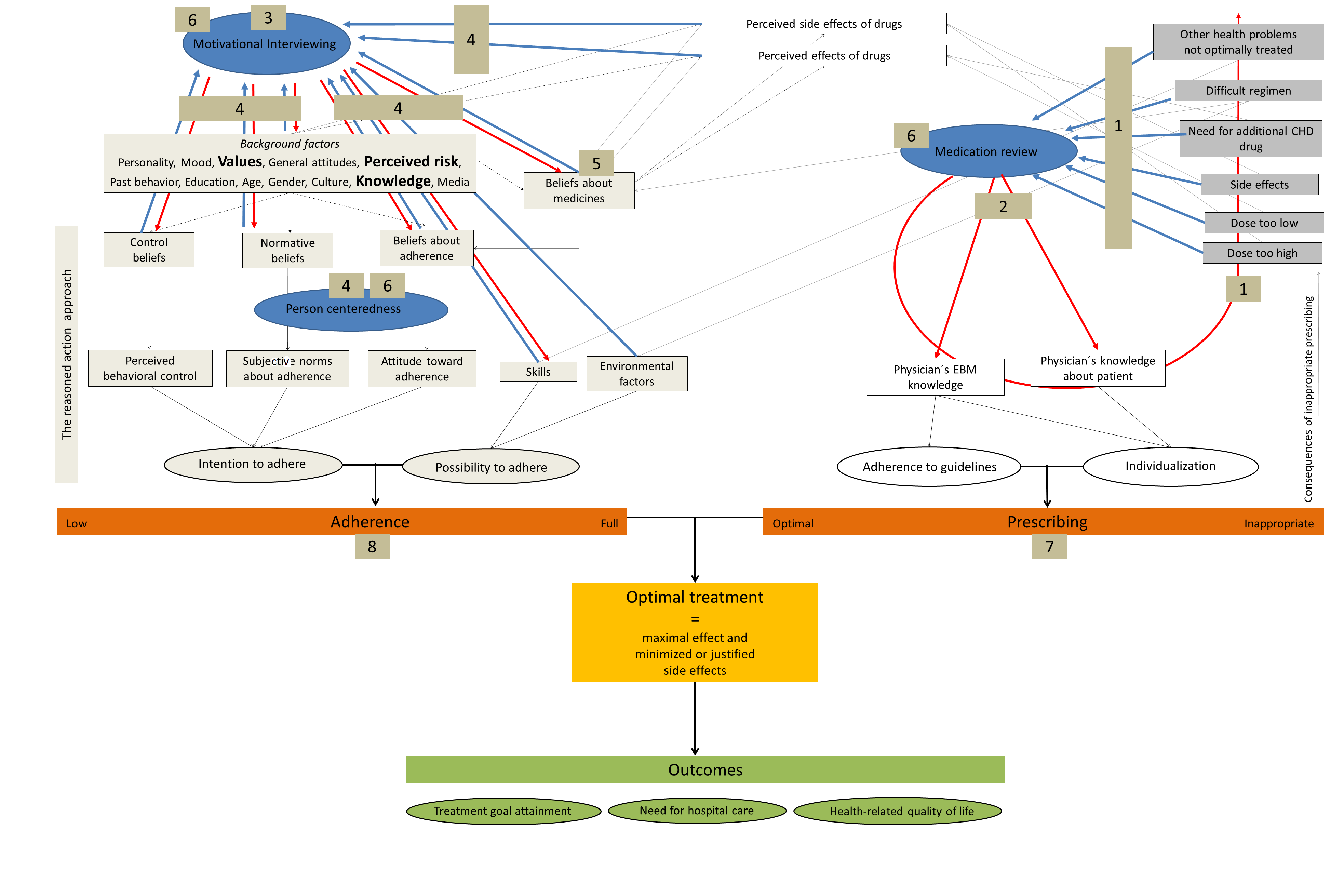

Supplement: Multimedia Appendix 2 [file resprot_v7i1e21_app2.png]
